# Mitochondrial genomes of *Anopheles arabiensis*, *An. gambiae* and *An. coluzzii* show no clear species division

**DOI:** 10.12688/f1000research.13807.2

**Published:** 2019-03-15

**Authors:** Mark J. Hanemaaijer, Parker D. Houston, Travis C. Collier, Laura C. Norris, Abdrahamane Fofana, Gregory C. Lanzaro, Anthony J. Cornel, Yoosook Lee

**Affiliations:** 1 Vector Genetics Laboratory, Department of Pathology, Microbiology and Immunology, University of California Davis , Davis, CA, 95616, USA; 2 Malaria Research and Training Center, University of Bamako, Bamako, E2528, Mali; 3 Mosquito Control Research Laboratory, Kearney Agricultural Center, Department of Entomology and Nematology, University of California Davis, Davis, CA, 93648, USA

**Keywords:** Mitogenome, species identification, Africa, malaria vector, mosquitoes, Anopheles, single nucleotide polymorphisms, phylogenomics

## Abstract

Here we report the complete mitochondrial sequences of 70 individual field collected mosquito specimens from throughout Sub-Saharan Africa. We generated this dataset to identify species specific markers for the following *Anopheles* species and chromosomal forms: *An. arabiensis*, *An. coluzzii* (The *Forest* and *Mopti* chromosomal forms) and *An. gambiae *(The *Bamako* and *Savannah* chromosomal forms).  The raw Illumina sequencing reads were mapped to the NC_002084 reference mitogenome sequence. A total of 783 single nucleotide polymorphisms (SNPs) were detected on the mitochondrial genome, of which 460 are singletons (58.7%). None of these SNPs are suitable as molecular markers to distinguish among *An. arabiensis*, *An. coluzzii* and *An. gambiae* or any of the chromosomal forms. The lack of species or chromosomal form specific markers is also reflected in the constructed phylogenetic tree, which shows no clear division among the operational taxonomic units considered here.

## Introduction

Historically, mtDNA sequence has been used in taxonomy as a source of species diagnostic markers ( Cronin *et al.* (1991); De Barba *et al.* (2014); Pegg *et al.* (2006)) or in population genetics and evolutionary studies ( Fu *et al.* (2013); Harrison (1989); Llamas *et al.* (2016)). One advantage of using mitochondrial over nuclear DNA for such studies is that the mutation rate of mtDNA is about 10 times faster than nuclear DNA ( Brown *et al.* (1979); Haag-Liautard *et al.* (2008)), hence amplifying the evolutionary trajectory of populations and species. In addition, mtDNA is easy to amplify, because there are more copies of mitochondrial DNA relative to nuclear DNA. Also, universal primers can be applied to a wide range of species. Widely used universal primers target the cytochrome b and cytochrome oxidase 1 genes ( Tahir *et al.* (2016)), because both have conserved and highly variable regions. In addition to these, other genes as described in De Mandal *et al.* (2014), can also be used as markers. However, phylogenetic trees based on mtDNA can deviate from the ones that are derived from nuclear DNA ( Phillips *et al.* (2013); Shaw (2002); Sota & Vogler, 2001).

The *Anopheles gambiae* species complex consists of eight morphologically identical species that can only be distinguished with molecular markers ( Scott *et al.* (1993); Coetzee *et al*., 2013) or, for some of the species, by cytological examination of polytene chromosomes ( Green, 1972; Pombi *et al*., 2008). The currently used molecular markers to distinguish between *An. coluzzii* and *An. gambiae* ( Lee *et al*., 2014) are located within genomic islands of divergence located proximal to the centromeres ( Turner *et al*. (2005)). Monitoring additional species-specific markers on mitochondrial DNA (mtDNA) could increase the ease of application and accuracy of species detection assays. In addition, mtDNA markers could enhance our understanding of divergence times among taxa within the complex.

Previous studies showed that there is a high amount of interspecific gene flow in mtDNA between *An. coluzzii*, *An. gambiae* and *An. arabiensis* specimens ( Besansky *et al*., 2003; Besansky *et al*., 1997; Donnelly *et al*., 2004). Although these data suggested no evidence for clear species division among the various species, the studies only focused on the ND5 loci ( Besansky *et al*., 2003; Donnelly *et al*., 2004) or included also cytochrome *b* and *ND1* loci ( Besansky *et al*., 1997). In our study we use the complete mitogenome for comparison, which would make the analysis more robust. In addition, we specifically included the different chromosomal forms in our analysis. These chromosomal forms are genetically diverged from each other and display strong assortative mating in the *An. gambiae* chromosomal forms ( Touré *et al*., 1998). The *An. coluzzii* chromosomal forms differ from each other in their ecology: *An. coluzzii*-Mopti is found in dry areas whereas the *An. coluzzii*-Forest restrtict themselves to a wet climate ( Lee *et al*., 2009).

In this study we wished to identify species-specific markers within the mtDNA for *Anopheles arabiensis*, *An. coluzzii* and *An. gambiae*, including among the chromosomal forms currently subsumed under the designations *An. gambiae* and *An. coluzzii*, with the goal of adding these to our existing *Anopheles* species detection assay ( Lee *et al.* (2014)). We sequenced the whole mitogenomes of 70 individual mosquito specimens collected throughout Sub-Saharan Africa. The raw Illumina sequencing reads were mapped to the AgamP4 reference sequence, which included both nuclear and mitochondrial sequences. We explore the relationship among *An. arabiensis, An. coluzzii*, *An. gambiae* and four of the sub-specific chromosomal form mitogenome sequences.

## Methods

### Sample collection

*Anopheles arabiensis* raw Illumina sequencing reads were obtained from our previous study ( Marsden *et al.* (2014)). These included 20 female *An. arabiensis* mosquitoes which were collected indoors in houses using mouth aspirators from three villages in Tanzania in 2012 (Lupiro ((-8.38000°N, 36.66912°W), Sagamaganga (-8.06781°N, 36.80207°W), and Minepa (-8.25700°N, 36.68163°W) in the Kilombero Valley) and 4 samples from Cameroon collected in 2005 (9.09957°N, 13.72292°W). The DNA was extracted from the head and thorax of each mosquito species and *An. arabiensis* mosquitoes were identified using Scott primers ( Scott *et al*., 1993)). The adult *An. gambiae* and *An. coluzzii* samples were collected indoors using mouth aspirators in Kela, Mali (11.88683°N, -8.44744°W) in 2012 and Mutengene, Cameroon (4.0994°N, 9.3081°W) in 2011. We subdivided the *An. coluzzii* specimen into the *Forest* and *Mopti* chromosomal forms. Similarly, we did this for the *An. gambiae Savannah* and *Bamako* chromosomal forms. We examined the polytene chromosome to characterize the chromosomal forms as in Lanzaro & Lee, 2013 and used the same definitions. The results of chromosome determination are listed in Table 1. The *An. quadriannulatus* mosquito, used as an outgroup for the phylogenetic analysis, was collected as larvae in the Shingwidzi area (23.1160°S 31.3752°E) in South Africa in 2015 and was reared to adult.

**Table 1.** List of detected chromosomal inversions to detect chromosomal forms of *An. coluzzii* and *An. gambiae* according Toure and co-workers ( Touré *et al.*, 1998). ‘2’ represents homozygous for the inversion, ‘1’ heterozygous for the inversion and ‘-‘ for homozygous for the standard arrangement.

Banked ID	Chromosomal Form	2La	2Rb	2Rc	2Rd	2Rj	2Ru
11MUTE470	*An. coluzzii*-Forest	-	-	-	-	-	-
11MUTE472	*An. coluzzii*-Forest	-	-	-	-	-	-
11MUTE476	*An. coluzzii*-Forest	-	-	-	-	-	-
11MUTE477	*An. coluzzii*-Forest	-	-	-	-	-	-
11MUTE479	*An. coluzzii*-Forest	-	-	-	-	-	-
11MUTE480	*An. coluzzii*-Forest	-	-	-	-	-	-
11MUTE483	*An. coluzzii*-Forest	-	-	-	-	-	-
11MUTE487	*An. coluzzii*-Forest	-	-	-	-	-	-
11MUTE490	*An. coluzzii*-Forest	-	-	-	-	-	-
11MUTE491	*An. coluzzii*-Forest	-	-	-	-	-	-
11MUTE493	*An. coluzzii*-Forest	-	-	-	-	-	-
2012KELA022	*An. coluzzii*-Mopti	1	1	1	-	-	-
2012KELA024	*An. coluzzii*-Mopti	2	1	1	-	-	-
2012KELA046	*An. coluzzii*-Mopti	2	1	1	-	-	-
2012KELA085	*An. coluzzii*-Mopti	2	2	2	-	-	-
2012KELA087	*An. coluzzii*-Mopti	1	2	2	-	-	-
2012KELA088	*An. coluzzii*-Mopti	2	-	-	-	-	1
2012KELA099	*An. coluzzii*-Mopti	2	-	-	-	-	1
2012KELA112	*An. coluzzii*-Mopti	2	2	2	-	-	-
2012KELA161	*An. coluzzii*-Mopti	2	-	-	-	-	1
2012KELA210	*An. gambiae*-Savannah	2	2	-	-	-	-
2012KELA214	*An. gambiae*-Bamako	2	-	2	-	2	2
2012KELA219	*An. gambiae*-Bamako	2	-	2	-	2	2
2012KELA228	*An. gambiae*-Savannah	2	2	-	-	-	-
2012KELA233	*An. gambiae*-Savannah	2	2	-	-	-	-
2012KELA234	*An. gambiae*-Savannah	1	2	-	-	-	-
2012KELA239	*An. gambiae*-Bamako	2	1	2	-	2	2
2012KELA240	*An. gambiae*-Bamako	2	1	2	-	2	2
2012KELA244	*An. gambiae*-Bamako	2	-	2	-	2	2
2012KELA285	*An. gambiae*-Savannah	2	2	-	-	-	-
2012KELA321	*An. gambiae*-Savannah	2	2	-	-	-	-
2012KELA334	*An. gambiae*-Savannah	2	2	-	-	-	-
2012KELA348	*An. gambiae*-Savannah	2	2	-	-	-	-
2012KELA367	*An. gambiae*-Bamako	2	1	2	-	2	2
2012KELA400	*An. coluzzii*-Mopti	2	-	-	-	-	2
2012KELA406	*An. gambiae*-Bamako	2	-	2	-	2	2
2012KELA409	*An. gambiae*-Savannah	2	2	-	-	-	-
2012KELA420	*An. coluzzii*-Mopti	2	-	-	-	-	2
2012KELA423	*An. coluzzii*-Mopti	2	2	2	-	-	-
2012KELA443	*An. gambiae*-Bamako	2	1	2	-	2	2
2012KELA457	*An. gambiae*-Bamako	2	-	2	-	2	2
2012KELA458	*An. coluzzii*-Mopti	2	-	-	-	-	2
2012KELA467	*An. gambiae*-Bamako	2	-	2	-	2	2
2012KELA468	*An. gambiae*-Savannah	2	1	-	-	-	-
2012KELA481	*An. gambiae*-Bamako	2	2	2	-	2	2
2012KELA496	*An. coluzzii*-Mopti	2	1	-	-	-	-
2012KELA651	*An. gambiae*-Bamako	2	2	2	-	2	2
2012KELA812	*An. gambiae*-Savannah	2	1	-	-	-	-

### Genome sequencing

Sequencing methods for *An. arabiensis* samples are as described in Marsden *et al.* (2014). In short, individually barcoded Illumina paired-end sequencing libraries, with insert sizes of 320-400 basepairs (bp) using NEXTflex Sequencing kits (NOVA-5144) and barcodes (NOVA-514102)(Bio Scientific, Austin, TX, USA), were sequenced on an Illumina HiSeq2000 (Illumina, San Diego, CA, USA) with 100-bp paired-end reads using twelve samples per lane. For the *An. coluzzii* and *An. gambiae* samples we used the same methods as described in Norris *et al.* (2015) and Main *et al.* (2015). For the latter species, libraries were created using the Nextera DNA Sample Preparation Kit (FC-121-1031) and TruSeq dual indexing barcodes (FC-121-103)(Illumina) and the samples were sequenced on an Illumina HiSeq2500 with 100-bp paired end reads. We sequenced the whole genome, but only mapped the raw sequences to the NC_002084 reference mitogenome sequence.

### Data analysis

De-multiplexed raw reads were trimmed using Trimmomatic ( Bolger *et al.* (2014)) version 0.36 and mapped to the mitogenome reference sequence of *An. gambiae* (Genbank accession number = NC_002084 ( Beard *et al.* (1993))). Freebayes (v1.0.1) ( Garrison & Marth, 2013) was used for mitochondrial variant calling assuming single ploidy and without population prior. Mapping statistics were calculated using qualimap version 2.2 ( Okonechnikov *et al.* (2016)) and the data is represented in Table 2. Following the recommendation of Crawford and Lazarro ( Crawford & Lazzaro, 2012), we used a minimum depth of 8 to call variants for each individual. Between positions 1-13,470bp of the mitogenome, we obtained consistently high quality reads for all samples, which were used for further analysis. An AT-rich region located between 13,471 and 15,388 suffers from low or zero coverage for sequences generated with the Nextera library preparation kit. Therefore, we excluded these regions from further analysis. The Vcf2fasta program ( Danecek *et al.* (2011)) was used to extract mitogenome sequences from vcf file to fasta format. Geneious version 10.1.3 was used for mitogenome alignments. The phylogenetic tree was generated using PhyloBayes MPI ( Lartillot *et al*., 2013) using the CAT-GTR model on the genomic sequences, which is shown to give similar results compared to amino acid sequences ( Foster *et al*., 2017). We ran the program twice for over 30000 iterations. Max difference between the two runs was 0.045 and minimum effective size was > 100 and created a consensus tree that we visualized in Geneious version 10.1.3. We used scikit-allel (v1.1.9), a software package for Python ( Miles & Harding (2017)), to identify species specific markers.

**Table 2.** List of samples that are used for the study. Mapped reads indicates the reads that are mapped to the reference genome. Mean coverage indicates the average depth of reads on the mitochondrial DNA and standard deviation indicates the coverage deviation across the mitochondrial DNA.

Species	Banked_id	Year	Country	Village	Mapped bases	Mean coverage	Standard deviation
*An. coluzzii-Forest*	11MUTE470	2011	Cameroon	Mutengene	4265836	277.7	144.5
*An. coluzzii-Forest*	11MUTE472	2011	Cameroon	Mutengene	1862892	121.3	23
*An. coluzzii-Forest*	11MUTE476	2011	Cameroon	Mutengene	2130531	138.7	50.5
*An. coluzzii-Forest*	11MUTE477	2011	Cameroon	Mutengene	806611	52.5	16.7
*An. coluzzii-Forest*	11MUTE480	2011	Cameroon	Mutengene	804015	52.3	21
*An. coluzzii-Forest*	11MUTE483	2011	Cameroon	Mutengene	1702247	110.8	42.9
*An. coluzzii-Forest*	11MUTE487	2011	Cameroon	Mutengene	812839	52.9	21.2
*An. coluzzii-Forest*	11MUTE490	2011	Cameroon	Mutengene	1882088	122.5	52.4
*An. coluzzii-Forest*	11MUTE491	2011	Cameroon	Mutengene	1422997	92.6	46.6
*An. coluzzii-Forest*	11MUTE493	2011	Cameroon	Mutengene	627590	40.9	17.3
*An. coluzzii-Mopti*	12KELA022	2012	Mali	Kela	3695920	240.6	64.4
*An. coluzzii-Mopti*	12KELA024	2012	Mali	Kela	574282	37.4	30.8
*An. coluzzii-Mopti*	12KELA046	2012	Mali	Kela	4152520	270.3	87.2
*An. coluzzii-Mopti*	12KELA085	2012	Mali	Kela	10883282	708.4	345
*An. coluzzii-Mopti*	12KELA087	2012	Mali	Kela	3351158	218.1	79.8
*An. coluzzii-Mopti*	12KELA088	2012	Mali	Kela	1704283	110.9	91.3
*An. coluzzii-Mopti*	12KELA099	2012	Mali	Kela	349531	22.8	11
*An. coluzzii-Mopti*	12KELA112	2012	Mali	Kela	8550102	556.5	198.2
*An. coluzzii-Mopti*	12KELA161	2012	Mali	Kela	33794208	2199.7	629.3
*An. gambiae-Savannah*	12KELA210	2012	Mali	Kela	3007375	195.8	53.3
*An. gambiae-Bamako*	12KELA214	2012	Mali	Kela	26441050	1721.1	566.4
*An. gambiae-Bamako*	12KELA219	2012	Mali	Kela	3617355	235.5	130.2
*An. gambiae-Savannah*	12KELA228	2012	Mali	Kela	7783776	506.7	262.8
*An. gambiae-Savannah*	12KELA233	2012	Mali	Kela	7827363	509.5	138.6
*An. gambiae-Savannah*	12KELA234	2012	Mali	Kela	6721204	437.5	205.9
*An. gambiae-Bamako*	12KELA239	2012	Mali	Kela	6683521	435	126.4
*An. gambiae-Bamako*	12KELA240	2012	Mali	Kela	15131480	984.9	270.8
*An. gambiae-Bamako*	12KELA244	2012	Mali	Kela	12851754	836.5	306.5
*An. gambiae-Savannah*	12KELA285	2012	Mali	Kela	407888	26.6	119.8
*An. gambiae-Savannah*	12KELA321	2012	Mali	Kela	1034014	67.3	43.8
*An. gambiae-Savannah*	12KELA334	2012	Mali	Kela	20949015	1363.6	400.4
*An. gambiae-Savannah*	12KELA348	2012	Mali	Kela	12053890	784.6	280.9
*An. gambiae-Bamako*	12KELA367	2012	Mali	Kela	12109235	788.2	240.1
*An. coluzzii-Mopti*	12KELA400	2012	Mali	Kela	13707820	892.3	398.2
*An. gambiae-Bamako*	12KELA406	2012	Mali	Kela	17605437	1146	463.2
*An. gambiae-Savannah*	12KELA409	2012	Mali	Kela	10526480	685.2	259.1
*An. coluzzii-Mopti*	12KELA420	2012	Mali	Kela	31785953	2069	845.5
*An. gambiae-Bamako*	12KELA443	2012	Mali	Kela	25740781	1675.5	669.1
*An. gambiae-Bamako*	12KELA457	2012	Mali	Kela	1360654	88.6	36.6
*An. coluzzii-Mopti*	12KELA458	2012	Mali	Kela	153686	10	10.4
*An. gambiae-Bamako*	12KELA467	2012	Mali	Kela	10499093	683.4	249.1
*An. gambiae-Savannah*	12KELA468	2012	Mali	Kela	10315033	671.4	197.1
*An. gambiae-Bamako*	12KELA481	2012	Mali	Kela	20308589	1321.9	307.6
*An. coluzzii-Mopti*	12KELA496	2012	Mali	Kela	2975297	193.7	162.9
*An. gambiae-Bamako*	12KELA651	2012	Mali	Kela	376689	24.5	11.3
*An. gambiae-Savannah*	12KELA812	2012	Mali	Kela	799071	52	29.3
*An. arabiensis*	12LUPI001	2012	Tanzania	Lupiro	2843317	185.1	34.9
*An. arabiensis*	12LUPI007	2012	Tanzania	Lupiro	6288802	409.3	40
*An. arabiensis*	12LUPI024	2012	Tanzania	Lupiro	6328898	412	78.5
*An. arabiensis*	12LUPI056	2012	Tanzania	Lupiro	5440256	354.1	39.2
*An. arabiensis*	12LUPI059	2012	Tanzania	Lupiro	39721262	2585.5	801.8
*An. arabiensis*	12LUPI071	2012	Tanzania	Lupiro	3433158	223.5	59.2
*An. arabiensis*	12LUPI074	2012	Tanzania	Lupiro	10096062	657.2	100.5
*An. arabiensis*	12LUPI082	2012	Tanzania	Lupiro	5732773	373.2	69.6
*An. arabiensis*	12MINE001	2012	Tanzania	Minepa	7768923	505.7	66.9
*An. arabiensis*	12MINE040	2012	Tanzania	Minepa	2784428	181.2	54.9
*An. arabiensis*	12MINE100	2012	Tanzania	Minepa	10753877	700	93.9
*An. arabiensis*	12MINE101	2012	Tanzania	Minepa	5684230	370	41.9
*An. arabiensis*	12MINE105	2012	Tanzania	Minepa	1526829	99.4	32.8
*An. arabiensis*	12MINE111	2012	Tanzania	Minepa	5578562	363.1	76.3
*An. arabiensis*	12SAGA066	2012	Tanzania	Sagamaganga	12745079	829.6	142.3
*An. arabiensis*	12SAGA107	2012	Tanzania	Sagamaganga	14460217	941.2	259.2
*An. arabiensis*	12SAGA131	2012	Tanzania	Sagamaganga	15333239	998.1	282.9
*An. arabiensis*	12SAGA133	2012	Tanzania	Sagamaganga	3792945	246.9	62.5
*An. arabiensis*	12SAGA134	2012	Tanzania	Sagamaganga	2439101	158.8	34.5
*An. arabiensis*	12SAGA141	2012	Tanzania	Sagamaganga	3130504	203.8	33.3
*An. arabiensis*	05OKJ017	2005	Cameroon	Ourodoukoudje	9041052	588.5	78.8
*An. arabiensis*	05OKJ042	2005	Cameroon	Ourodoukoudje	148752684	9682.5	785.7
*An. arabiensis*	05OKJ045	2005	Cameroon	Ourodoukoudje	35514980	2311.7	262.8
*An. arabiensis*	05OKJ070	2005	Cameroon	Ourodoukoudje	22847478	1487.2	400.5

Aligned FASTA file of mitogenome samples Click here for additional data file. Copyright: © 2019 Hanemaaijer MJ et al. 2019 Data associated with the article are available under the terms of the Creative Commons Zero "No rights reserved" data waiver (CC0 1.0 Public domain dedication).

## Results and Discussion

We identified a total of 783 single nucleotide polymorphisms (SNPs) over the entire mitogenome. The majority of these (58.7%) were singletons (found on one of the 70 mitogenomes). We did not identify any SNPs unique to the species or chromosomal forms ( Supplementary Table S1) and therefore conclude that mtDNA is not suitable for *Anopheles gambiae* complex species identification.

**Figure 1.**
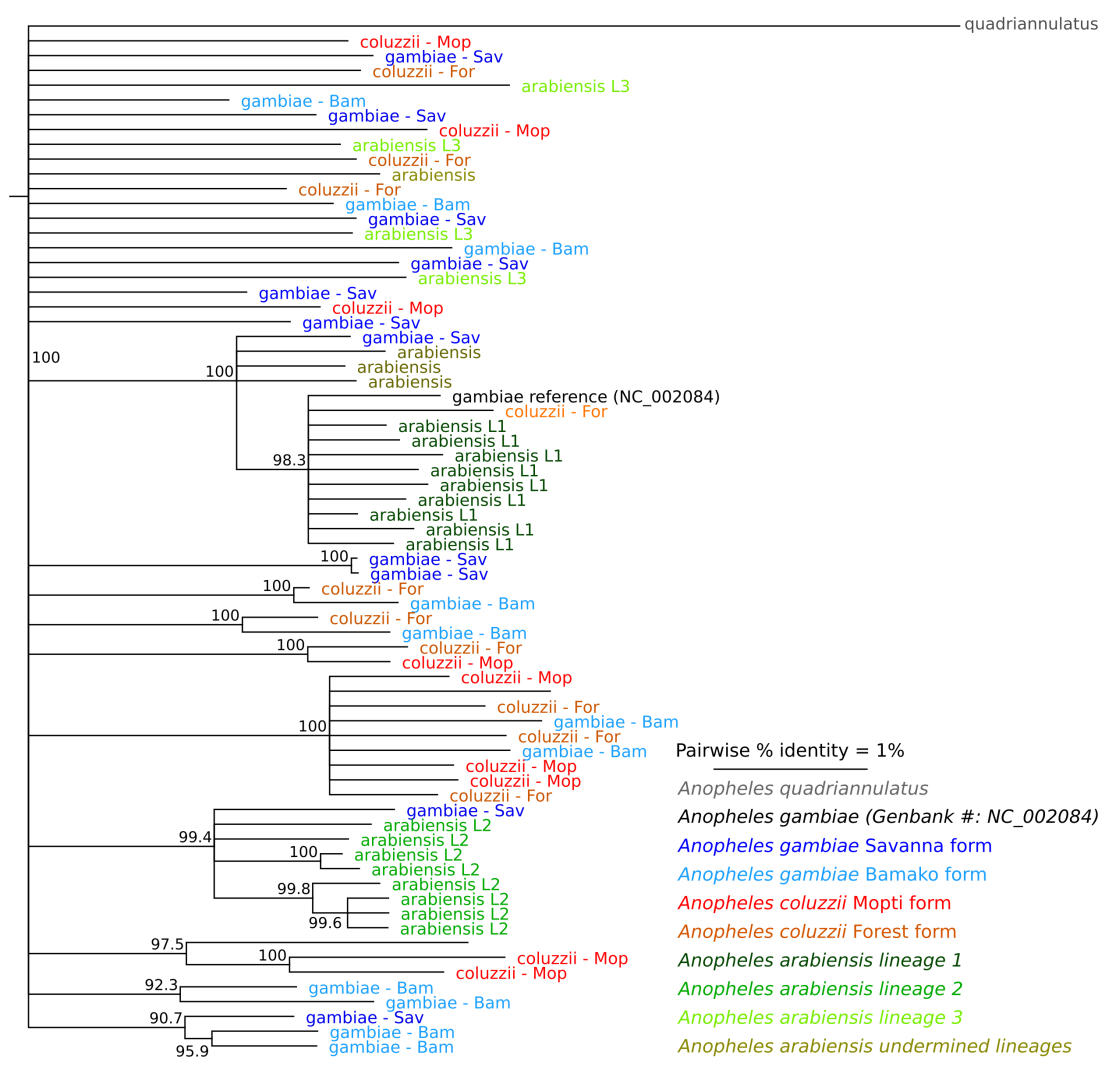
Phylogenetic tree inferred from mtDNA genome sequence data. The phylogenetic tree fails to reveal a clear division of the operational taxonomic units included in this analysis. Colors indicate the species or chromosomal form and numbers at the branches indicate the accuracy of the inferred branches on a scale of 0–1, where 1 represents the highest confidence. The three *An. arabiensis* lineages are previously reported by Maliti and co-workers ( Maliti *et al*., 2016).

The lack of species-specific markers is also reflected in the phylogenetic tree ( Figure 1). *An. arabiensis*, *An. coluzzii* and *An. gambiae* did not cluster separately, which is consistent with previous reports that compared mitochondrial genome sequence data from specimens originating from Kenya, Senegal and South Africa ( Besansky *et al.* (1997)) and Burkina Faso, Cameroon, Kenya, Mali, South Africa, Tanzania and Zimbabwe ( Fontaine *et al.* (2015), Supplemental material).

Our data may indicate that there is no divergent selection in mitogenome among *An. gambiae* complex. Since mitochondrial genomes have a higher (1–10 times) substitution rate than nuclear genomes ( Havird & Sloan, 2016; Lynch & Walsh, 2007), one might expect some level of divergence in the mitogenome in the absence of selection if the taxa have been separated by reproductive barrier even if they are in sympatry just as people have observed in nuclear genome. Therefore, our data showing lack of any species-specific markers on the mitogenome may due to the results of episodic hybridizations occurred between two species. Of note, 36 of the samples that we used in our study originated from Kela (Mali). Kela is located near the village of Selinkenyi, where previous studies have shown a history of hybridization and introgression between *An. gambiae* and *An. coluzzii* ( Lee *et al.* (2013); Main *et al.* (2015); Norris *et al.* (2015)), which may have resulted in shared polymorphisms in their mitochondrial genomes. Shared polymorphisms in their mitochondrial genomes, where history has not been reported, also appeared to have occurred in Mutengene (Cameroon), where both *An. gambiae* and *An. coluzzii* occur sympatrically. Hybridization between either *An. coluzzii* or *An. gambiae* with *An. arabiensis* yields sterile males ( Slotman *et al.* (2004)), but phylogenomic analysis of these species show patterns of introgression between all of them ( Fontaine *et al.* (2015)), which could be the reason that we do not find any species-specific markers on the mitogenome. Our mitochondrial genome study does not provide conclusive evidence for hybridization and introgression among the taxa under study. However, our data suggest that this is a possibility and it would be consistent with results reported by ( Fontaine *et al*., 2015) and ( Besansky *et al*., 1997). Future modeling work may illuminate the likely contribution of different evoluationary forces that shapes mitogenome and nuclear genome evolution.

## Data availability

The data referenced by this article are under copyright with the following copyright statement: Copyright: © 2019 Hanemaaijer MJ et al.

Data associated with the article are available under the terms of the Creative Commons Zero "No rights reserved" data waiver (CC0 1.0 Public domain dedication). 

Aligned sequences were submitted to the National Center for Biotechnology Information (NCBI) Accession number: MG930826 - MG930896

Dataset 1. Aligned FASTA file of mitogenome samples 10.5256/f1000research.13807.d192892 ( Hanemaaijer *et al*., 2018)

## Supplementary material

**Supplementary Table S1. List of SNP variants in the different Anopheles species and chromosomal forms.**
