# Supplementary material for: Mitochondrial genomes of Anopheles arabiensis, An. gambiae and An. coluzzii show no clear species division
Source: F1000Res. 2019 Mar 15;7:347. Originally published 2018 Mar 21. [Version 2] doi: 10.12688/f1000research.13807.2 (PMC6489993; doi:10.12688/f1000research.13807.2)
Supplement: Peer review history

# Peer review: Reviewer response for version 2

**DOI:** 10.5256/f1000research.20094.r45747

**Published:** 2019-04-29

**Authors:** Maria Anice Mureb Sallum

**Affiliations:** 1 Department of Epidemiology, Faculty of Public Health, University of São Paulo, São Paulo, Brazil

The revised version is suitable for publication and previously mentioned concerns have been clarified. The mitogenome annotation was well done, and the phylogenetic analyses were adequate. The writing is clear and correct. This work is an important contribution to our knowledge of the mitochondrial genome of the Anophelinae species complexes.

I have read this submission. I believe that I have an appropriate level of expertise to confirm that it is of an acceptable scientific standard.

# Peer review: Reviewer response for version 1

**DOI:** 10.5256/f1000research.15009.r33371

**Published:** 2018-05-14

**Authors:** Maria Anice Mureb Sallum

**Affiliations:** 1 Department of Epidemiology, Faculty of Public Health, University of São Paulo, São Paulo, Brazil

**General comment**

 Phylogenetic analysis need to be improved, and the choice for NJ methods and JC model, justified in the article. There are several programs that have been largely employed for phylogenetic analysis, including for mitogenome data. The paper authored by Foster et al. ^1^ contains useful information about analyses that have been carried out for inferring phylogenetic relationships within Anophelinae mosquitoes. I strongly suggest authors to verify how analyses were done.

**Sample collection**

**Authors - **“The An. gambiae and An. coluzzii samples were collected as resting adults using mouth aspirators in Kela, Mali (11.88683°N, -8.44744°W) in 2012 and Mutengene, Cameroon (4.0994°N, 9.3081°W) in 2011.”

**Comment -** Can you please give more details the micro environment where your specimens of An. gambiae and An. coluzzii were resting?

**Authors -** “Similarly, we did this for the An. gambiae Savannah and Bamako chromosomal forms. We used the same definitions and methods to characterize the chromosomal forms as in Lanzaro & Lee, 2013.”

**Comment -** It is not clear to me if you examined the polytene chromosome of each specimen you identified as the Savannah, Bamako, Forest and Mopti forms. Please clarify.

**Genome sequencing**

**Authors -** “For the An. coluzzii and An. gambiae samples we used the same methods as described in Norris et al. (2015) and Main et al. (2015). For the latter species, libraries were created using the Nextera DNA Sample Preparation Kit (FC-121-1031) and TruSeq dual indexing barcodes (FC-121-103) (Illumina) and the samples were sequenced on an Illumina HiSeq2500 with 100-bp paired end reads.”

**Comment -** Please add a short sentence to clarify if you sequenced the whole genome and from the full sequence data you obtained the positions 1-13,470 of the mitogenome.

**Data analysis**

**Authors -** “The phylogenetic tree was generated using the Jukes-Cantor genetic distance model and Neighbor-Joining tree methods available in Geneious version 10.1.3.”

**Comment - **Authors should clarify their choice for sequence analysis. The Geneious software has been developed for editing and aligning DNA / amino acid sequences. There are several softwares, which have been largely used to infer phylogenetic relationships. I suggest authors to refining and improving the phylogenetic analysis using appropriate programs and models that have been chosen for the mitogenome data you have at hand.

I have read this submission. I believe that I have an appropriate level of expertise to confirm that it is of an acceptable scientific standard, however I have significant reservations, as outlined above.

## References

[1] FosterPGde OliveiraTMPBergoESConnJESant'AnaDCNagakiSSNiheiSLamasCEGonzálezCMoreiraCCSallumMAM: Phylogeny of Anophelinae using mitochondrial protein coding genes. *R Soc Open Sci*.2017;4(11) : 10.1098/rsos.170758 170758 10.1098/rsos.170758 29291068PMC5717642

# Peer review: Reviewer response for version 1

**DOI:** 10.5256/f1000research.15009.r32266

**Published:** 2018-04-30

**Authors:** Beniamino Caputo, Verena Pichler

**Affiliations:** 1 Department of Public Health and Infectious Diseases, Sapienza University of Rome, Rome, Italy; 2 Department of Public Health & Infectious Diseases, Sapienza University of Rome, Rome, Italy

**General comments**

 The present research note entitled:” Mitochondrial genomes of *Anopheles arabiensis*, *An. gambiae* and *An. coluzzii* show no clear species division” is well analysed, reported and written. As already reported in previous study the submitted manuscript suggested the absence of any species-specific differences in the mitogenome of the three species examined. Although the manuscript is not innovative and the research is not based on any previous evidence, the present note confirms previous suggestions by examining the whole mitogenome of 70 specimens from field specimens and find the lack of species or chromosomal form specific markers.

**Title and abstract**

 Title and abstract are appropriate and summarize well the content of the article.

**Introduction**

 The introduction gives a good description of the aims of the present study, although I would have added some references to previous studies performed on mtDNA of the examined species (for example Besansky 1997) and why you expected to obtain different results compared to previous studies.

 Please revise also:

 “morphologically identical species that can only be distinguished with molecular markers”  (Scott *et al., *1993; Coetzee et al., 2013)

 The currently used molecular markers are located within genomic islands of divergence located proximal to the centromeres ( Lee et al. (2014); Turner et al. (2005)) please rephrase the citation and refer it only to detect genomic differences between *An.gambiae* e and *An.coluzzii*.

 Please insert a sentence about chromosomal forms of *An.gambiae.*

**Methods**

 Please specified the method for collecting *An. arabiensis* as you already described for *An.gambiae* (e.g. indoor specimens, mouth aspirators, PSC collections).

 Please insert a table with inversion polymorphism of chromosomal forms analyzed.

 Please add the source of the *An. quadriannulatus* specimens you included in the phylogenetic analysis.

**Results**

 Study design is well explained and results are given concisely.

 Please add in Table 2 also the number of specimens you included for each species in the analysis.

 Please add in Figure two an explanation of what “lineage” means for *An. arabiensis* specimens.

 Please give results (also without table or figure) for each country separately.

**Discussion**

 Discussion is very concise but deals with most major points of interest. We would just suggest to explain better the conclusion on possible introgression (the more plausible hypothesis) between taxa and to evaluate other possible explanations for the absence of fixed differences between species (e.g. absence for divergent selection, or evolutionary characherestic of mitogenomes).

We have read this submission. We believe that we have an appropriate level of expertise to confirm that it is of an acceptable scientific standard.
